# Identification and validation of a key genomic region on chromosome 6 for resistance to Fusarium stalk rot in tropical maize

**DOI:** 10.1007/s00122-022-04239-0

**Published:** 2022-10-22

**Authors:** Zerka Rashid, Veerendra Babu, Shyam Sundar Sharma, Pradeep Kumar Singh, Sudha Krishnan Nair

**Affiliations:** 1 International Maize and Wheat Improvement Center (CIMMYT), ICRISAT Campus, Patancheru, Greater, Hyderabad, 502324 Telangana India; 2 Maharana Pratap University of Agriculture and Technology (MPUAT), Udaipur, 313001 Rajasthan India; 3 Corteva Agriscience Seeds India Pvt Ltd., Madhapur, Hyderabad, 500081 Telangana India

## Abstract

**Key message:**

A key genomic region was identified for resistance to FSR at 168 Mb on chromosome 6 in GWAS and haplotype regression analysis, which was validated by QTL mapping in two populations.

**Abstract:**

Fusarium stalk rot (FSR) of maize is an economically important post-flowering stalk rot (PFSR) disease caused by *Fusarium verticillioides*. The pathogen invades the plant individually, or in combination with other stalk rot pathogens or secondary colonizers, thereby making it difficult to make accurate selection for resistance. For identification and validation of genomic regions associated with FSR resistance, a genome-wide association study (GWAS) was conducted with 342 maize lines. The panel was screened for FSR in three environments using standard artificial inoculation methodology. GWAS using the mixed linear model corrected for population structure and kinship was done, in which 290,626 SNPs from genotyping-by-sequencing were used. A total of 7 SNPs, five on chromosome 6 showing strong LD at 168 Mb, were identified to be associated with FSR. Haplotype regression analysis identified 32 haplotypes with a significant effect on the trait. In a QTL mapping experiment in two populations for validating the identified variants, QTLs were identified with confidence intervals having overlapped physical coordinates in both the populations on chromosome 6, which was closely located to the GWAS-identified variants on chromosome 6. It makes this genomic region a crucial one to further investigate the possibility of developing trait markers for deployment in breeding pipelines. It was noted that previously reported QTLs for other stalk rots in maize mapped within the same physical intervals of several haplotypes identified for FSR resistance in this study. The possibility of QTLs controlling broad-spectrum resistance for PFSR in general requires further investigation.

## Introduction

Due to the population and socioeconomic growth, the global food requirement is expected to be double the current demand by 2050 (Ray et al. 2013). Fulfilling this demand, developing countries, specifically, must double the production of staple cereals like rice, wheat and maize. Climate change has adversely affected the agricultural production throughout the world and subsequently it is distressing for the crops especially for the regions like East Africa (EA) and South Asia (SA) (Chivasa et al. 2021). Maize is an important crop in SA, where a large portion of maize (~70% of total volume) is used by the feed industry, which is the most important supplier for the protein demand (Shiferaw et al. 2011). Apart from feed, it is used as food and increasingly used in food processing industry for making additives and sweeteners (Prasanna 2018). The maize production and productivity in the Asian region have shown accelerated growth and is the second largest maize producer in the world with its 31% share in global maize production (Zaidi et al. 2018). But climate change effects have predicted to reduce the maize yield by 7.4% with every 1 °C rise in mean global temperature (Zhao et al. 2017). It is projected that climate change would reduce rain-fed maize yield in South Asia by an average of 3.3–6.4% in 2030 and 5.2–12.2% in 2050 and irrigated yield by 3–8% in 2030 and 5–14% in 2050 if current varieties were grown (Tesfaye et al. 2017). Apart from the direct effect of climate change on agricultural systems, the small holder farmers of SA will face the climate-induced risks like increased crop pests and diseases which will have an impact on crop yield (Ali et al. 2014). With the change in precipitation, temperature, humidity and wind, the life cycle of the plant pathogens will get affected, resulting in the changes in virulence (Prasanna et al. 2021). Among the maize diseases, stalk rots and ear rots are the diseases that are reported to have the highest impact with climate change (Prasanna et al. 2021). Common post-flowering stalk rots occurring in maize are Fusarium stalk rot (FSR) caused by *Fusarium verticillioides* (synonym, *Fusarium moniliforme* Sheldon), *Gibberella* stalk rot (GSR) caused by *Fusarium graminearum*, anthracnose stalk rot (ASR) caused by *Colletotrichum graminicola*, *Diplodia* stalk rot caused by *Diplodia maydis*, Late wilt caused by *Harpophora maydis* (previously known as *Cephalosporium maydis*) and Charcoal rot caused by *Macrophomina phaseolina*. These pathogens could occur in isolation or in combination, along with secondary colonizers and other abiotic stress-related factors to cause severe incidences of late-stage post-flowering stalk rots. As these diseases affect the crop at a much later stage in the crop life cycle, the economic impact of the disease is too high and hence is one of the biggest challenges faced by small and marginal farmers.

*Fusarium verticillioides;* synonym, *Fusarium moniliforme* Sheldon is one of the aggressive pathogens that causes seed rot, seedling disease, stalk and ear rot and reduction in maize yield globally (Williams, 2008). It also produces toxic compounds called fumonisins, in diseased and symptomless maize kernels which are responsible for leukoencephalomalacia**,** pulmonary oedema, liver disorders and cancer in animals (Nelson et al. 1993). *Fusarium* species are vertically transmitted to the next generation of plants via infection of seeds and horizontally transmitted to plants through saprophytic infection of plant debris in the soil and insect vectors (Bacon et al. 2001). Conidia and hyphae of *F. moniliforme* were reported to survive two winters in Kansas on sorghum stalks without losing pathogenicity (Manzo et al. 1984). The stalk rot pathogens thrive on good vegetative stage growth followed by stresses like drought, nutrient deficiencies, foliar diseases, insect and hail damage, high heat and prolonged cool and cloudy weather during flowering (Dodd 1980). *F verticillioides* can infect any part of the maize plant from beginning to end of the cropping season, and hence FSR is a systemic disease of maize which starts from roots and escalates to the aerial parts of the plants after flowering. It spreads through internodes and causes the disintegration of pith tissues which results in weakening of the plants, early dryness and eventually plant lodging resulting in severe yield loss. Infected stalks have characteristic pinkish to reddish discoloration of pith and vascular strands and the rotting affects the roots, crown and lower internodes (Khokkhar et al. 2014). The severity of shredding and breaking down of pith at nodes increases as the plant matures after pollination. The disease becomes widespread when there is water scarcity during and after flowering. Stalk rots are more severe and show high incidence with increased fertilization of soils, especially increased soil nitrogen increases disease incidence (Abney and Foley 1971). Increase in plant population increases disease severity and incidence, especially in susceptible entries (White 1999). The disease is prevalent in almost all the countries of south and southeast Asia like China, Cambodia, India, Indonesia, Nepal, Pakistan, Philippines, Thailand. FSR is estimated to reduce 18.7% in cob weight and 11.2% in 1000-grain weight in the infected plants (Cook 1978). The All India Coordinated Research Program on Maize (Maize AICRP, 2014) estimated 38% of the yield loss due to FSR, while in Nepal the yield loss was estimated up to 80% (Subedi et al. 2016) caused by FSR disease. In Northern USA, FSR caused the second greatest yield loss of corn (to a tune of 110.9 million bushels) after tar spot during the year 2021 (Muller et al. 2022).

To understand the genetics of host resistance to this complex disease, various researchers conducted genetic analysis and reported that it is a polygenic trait which is quantitatively inherited, with significant genotype × environmental interaction (Szoke et al. 2007, Khokhar et al. 2014) with predominantly additive gene action (Mir et al. 2018, Donahue et al. 1989) towards resistance to FSR. In seedling blight caused by the same pathogen, Lunsford et al. (1974, 1976) reported that additive gene effects and maternal effects are more important than dominant gene action in its inheritance. To develop varieties that incorporates traits like resistance to quantitative diseases like FSR, which delivers a higher rate of genetic gain, holistic breeding methods incorporating modern breeding tools and strategies must be used. Breeding efficiency for disease resistance could be significantly improved by using molecular markers in selection, where there is feasibility to expand the size of breeding populations, thereby increasing the selection intensity without increasing the phenotyping demands. Selections using trait markers identified from QTL (quantitative trait loci) or linkage mapping and genome-wide association mapping (GWAS) could be used apart from employing genome-wide markers for genomic selection (GS). Identification and fine-mapping of moderate to high effect genes/QTL also increase the accuracy of selection in marker-based breeding (Nair et al. 2015). QTLs have been mapped for resistance to PFSRs like Charcoal rot (Rashid et al. 2021), GSR (Yang 2010; Zhang et al. 2012; Ma et al. 2017) and ASR (Jung et al. 1994; Abad et al. 2006; Broglie et al. 2011). A study that mapped QTL for resistance to FSR in maize was reported by Salah et al (2016), they studied the trait at vegetative stage and not at post-flowering stage. Their analysis revealed four SSR markers and one STS marker linked to FSR resistance on chromosome 10. Genomic studies were reported on seedling rot of maize caused by *F. verticillioides* by various researchers. Septiani et al. (2019) conducted QTL mapping in a Multi-parent Advance Generation Intercross (MAGIC) population derived from temperate maize lines for seedling rot using paper towel method. They identified three QTLs for Fusarium seedling rot, two on chromosome 4 at 2.8 Mb and 241.8 Mb, and one QTL was identified on chromosome 5 at 169Mb. In a GWAS conducted by Stagnati et al. (2020) on 230 inbred lines, some of the SNPs identified for seedling rot co-localized within QTL intervals previously identified for Fusarium seed rot, Fusarium ear rot and fumonisin accumulation in maize. GWAS studies for resistance to FSR at post-flowering stage have not been reported yet, possibly because of the difficulty in conducting high-precision trials under artificial inoculation, or the risk of co-infection with other soil-borne stalk rot pathogens under natural infection, and less variability in disease responses in the germplasm for this disease. FSR is an economically important disease in the Asian region and considering that it is a late-stage disease, due to which there are implications in phenotypic selections in breeding populations, it is important to investigate the possibility of identifying genomic regions contributing for resistance to the disease that could aid in trait selection. Also, considering the fact that there are no research reports on comprehensive mapping of resistance loci for this trait, we conducted a GWAS and haplotype regression analysis in an association mapping panel from Asia-adapted germplasm from International Maize and Wheat Improvement Center (CIMMYT) to identify trait markers associated with FSR resistance. The genomic regions identified in GWAS were further validated through QTL mapping using two F_2:3_ mapping populations.

## Materials and methods

### Plant material

The CAAM panel assembled at CIMMYT, consists of 419 tropical/sub-tropical inbred lines bred in Asia and other tropical geographies and acclimatized to the Asian tropics. This panel involves inbred lines that were bred for resistance/tolerance to different stresses like drought, high moisture, high temperature and downy mildew. The lines are adapted to tropical, sub-tropical, lowland, mid-altitude and highland environments, and based on growing degree days (GDD) the lines are grouped into early, medium and late maturity groups. A subset of 342 inbred lines from this panel were included in GWAS.

For linkage mapping, two F_2:3_ populations were developed for validation of genomic regions identified in GWAS and HTR analysis. The first population FSR-1 developed from a cross between the resistant parent CML578, is an orange flint line derived from waterlogging stress-tolerant population in Asia. This line was also used as a resistant parent for QTL mapping for charcoal rot resistance (Rashid et al. 2021). In the second population FSR-2, the resistant parent CML329/MBRc2amF14-2-B*7 is a flint yellow line originated from insect-resistant population from Mexico. In both the populations CML474 was used as susceptible parent, which is an Asia-lowland adapted early line.

## Phenotypic evaluation

The GWAS panel was evaluated for FSR under artificial inoculation conditions at International Crop Research Institute for Semi-Arid Tropics (ICRISAT) campus at Hyderabad (17.53° N; 78.27° E.; 545 masl; 784 mm/year average rainfall) for two years and CIMMYT managed farm at Daulatabad for one year (17.53° N; 78.27° E.; 545 masl; 784 mm/year average rainfall). For linkage mapping analysis, two F_2:3_ mapping populations, FSR-1 and FSR-2, with population size of 256 and 166 entries, respectively, were evaluated at Daulatabad. All FSR evaluation trials were planted in two replications following the alpha lattice design. The row length was 2m with a spacing of 0.20 m between plant to plant and 0.75m between row to row. Standard agricultural practices were followed throughout the cropping season.

### Inoculum development and artificial Inoculation technique

*Fusarium verticillioides* samples were isolated from the previously infected maize stalks. Infected stalks were cut into 5–10 mm small pieces, washed with 0.6% sodium hypochlorite for 1 minute and rinsed with sterile distilled water for 3–4 times under aseptic conditions. Excess water was blot dried on sterile tissue paper and infected leaf pieces were placed on Petri plates carrying pure culture Potato Dextrose Agar (PDA). The plates were incubated at 28 °C for 3–5 days, the growing hyphal tips were transferred to PDA allowed to grow for 8-10 days at 28 °C, spores were isolated using single spore isolation method.

Toothpick method was followed for artificial inoculation of the trials (Lal and Singh, 1984). In this method, mass multiplication of *F. verticillioides* for artificial inoculation was done on wooden toothpicks by the method proposed by Jardine and Leslie (1992), with minor modifications. For inoculum multiplication, wooden toothpicks were dipped in tap water for 12 to 15 hours followed by air drying. Dried toothpicks (approximately 250) were packed in 250 ml glass bottles with 50 ml distilled water and were autoclaved at 15 lbs and 121 °C for 15 minutes. After sterilization, excess water was poured out of the glass bottles and potato dextrose broth (PDB) was added, followed by autoclaving at the same temperature and pressure regime. After cooling, freshly sub-cultured fungi were inoculated into bottles under aseptic conditions and incubated at 25 °C till the toothpicks were covered up with fungal growth (approximately 15 days). Colonized toothpicks were inserted into the stalks at the tassel emergence stage of the plants. This was accomplished by drilling a hole of 4-5 cm at 45° angle in the second internode (first elongated node) with an iron needle having wooden handle, where the toothpicks were introduced into the hole.

Disease severity was recorded by splitting the infected plants in each plot longitudinally at the harvest. Split stalks were individually scored on disease severity scale of 1–9 (Payak and Sharma 1983) with score 1–2 rated as highly resistant (HR), 2.1–4 as resistant (R), 4.1–6 as moderately resistant (MR) and > 6.1 were susceptible (S). In each row, at least 10 plants were inoculated, and each inoculated plant was scored to obtain a mean disease score for the plot.

### Phenotypic data analysis

All phenotypic data analyses were carried out in META-R software (Alvardo et al. 2015). To analyse single and multi-location trials mixed linear model was used where all the factors, genotypes, environments, interaction between genotype with environment and interaction with replication and environment were considered as random effects.

$${\text{Y}}_{{{\text{ijk}}}} = \, \mu \, + {\text{ g}}_{{\text{i}}} + {\text{ l}}_{{\text{j}}} + {\text{ b}}_{{{\text{kj}}}} + \, \varepsilon_{{{\text{ijk}}}}$$

where Y_ijk_ was the disease severity of the i^th^ genotype at the j^th^ environment in the k^th^ incomplete block, µ was an intercept term, g_i_ was the genetic effect of the i^th^ genotype, l_j_ was the effect of the j^th^ environment, b_kj_ was the effect of the k^th^ incomplete block at the j^th^ environment, and ε_ijk_ was the error term confounding with the genotype-by-environment interaction effect. Broad-sense heritability of the combined analysis across years was estimated as

$${\text{H}}^{{2}} = \, \sigma^{{2}}_{{\text{g}}} /\left( {\sigma^{{2}}_{{\text{g}}} + \, \sigma^{{2}}_{{{\text{ge}}}} /e + \, \sigma^{{2}}_{{\text{e}}} /er} \right)$$

where σ^2^_g_, σ^2^_ge_ and σ^2^_e_ are the genotypic, genotype-by-year interaction and error variance components, respectively, and e and r are the number of years and number of replicates within each year included in the corresponding analysis, respectively. For the CAAM panel and two F_2:3_ mapping populations, best linear unbiased predictions (BLUPs) were generated for each genotype using the META-R software.

### Genotyping of CAAM panel and mapping populations

Genomic DNA of the association mapping panel and two F_2:3_ mapping population were extracted from the leaves of 3-4 weeks old seedlings using the standard protocol followed in CIMMYT. Genotyping of the CAAM panel was carried out at Institute for Genomic Diversity, Cornell University, Ithaca, NY, USA using genotyping-by-sequencing (GBS) platform. The GBS libraries were constructed as reported by Elshire et al. 2011, and SNP calling was performed using TASSEL GBS pipeline described by Glaubitz et al. 2014. The partially imputed GBS SNP data had 955,690 genotypic data points (SNPs) across all the chromosomes was based on an algorithm that explores closest neighbour in small SNP windows across the whole genome, permitting 5% mismatch (Romay et al. 2013). A total 295,794 SNPs, that passed the quality control filtration criteria of call rate (CR) ≥ 0.7 to account for missing data and minor allele frequency (MAF) ≥ 0.05 were used in different models of GWAS analysis for FSR resistance.

For two mapping populations for the QTL mapping study, markers were selected across the 10 chromosomes from the Illumina Goldengate assay. The lines were genotyped with Kompetitive Allele Specific PCR (KASP) assays developed from sequences of random and GWAS-identified SNP markers at LGC Genomics, London. (https://www.biosearchtech.com/services/genotyping-services, Herts, UK). F_2:3_ mapping populations FSR-1 and FSR-2 were genotyped with a set of 272 and 268 markers, respectively, based on parental line polymorphism.

### GWAS and haplotype analysis

GWAS was carried out based on three models: (i) G-test in which only genotypic data (G) was used, (ii) General linear model (GLM) where genotypic data was corrected for structure (Q) using 10 principal components (PCs) and (iii) single locus mixed linear model (MLM) (Kang et al, 2010) where genotypic data was corrected for Q using 10 PCs and kinship matrix (K) to avoid false associations. In earlier GWAS studies in the CAAM panel, principal component analysis did not reveal a clear population structure with the first three principal components. The Scree plot plotted with all the eigenvalues suggested 10 principal components will be able to adjust the existing population structure (Rashid et al. 2018). A kinship matrix was estimated from identity-by-state distances matrix as executed in SNP & Variation Suite (Golden Helix, Inc., Bozeman, MT, www. goldenhelix.com) (SVS) Version_8.6.0, where IBS distance = (no. of markers IBS2) + 0.5 × (no. of markers IBS1) no. non-missing markers, and IBS1 and IBS2 are the states in which the two inbred lines share one or two alleles, respectively, at a marker (Bishop & Williamson 1990).

Additive models were used for testing the three models in SVS Version 8.6.0 The mixed association mapping model used was: Y = SNP*β + PC*α + K *μ + ε, where Y = response of the dependent variable (FSR Score), SNP = SNP marker (fixed effects), PC = principal component coordinate from the PCA (fixed effects), K = kinship matrix (random effects), α is the vector of PC, β and μ are the vectors of SNP and K, respectively, and ε is the error. Manhattan plots were used for visualization of GWAS results by plotting *P *values on logarithmic scale. The values are plotted in the linear order based on the chromosomal position of the SNPs in SVS version 8.6.0. Quantile–quantile plots (Q–Q plots) inferred the severity of inflation test statistics by plotting the observed versus expected negative log10 *P value* that were generated for all the models studied according to established procedures*.* Q–Q plots from the three models were represented in one plot using R-studio software (RStudio Team 2020).

Linkage disequilibrium (LD) was estimated based on pairwise r^2^ values between adjacent SNPs and physical distances between those SNPs as described in Rashid et al. 2020. Markers that were in linkage equilibrium with each other were determined based on SNP pruning with LD r^2^ threshold of 0.1 (window size 50, step size 50) and estimated as 126,120. The suggestive *P value* threshold to control the genome-wide type 1 error rate was estimated as 7.92 ×10^-6^ which was considered as the significance cut-off for the association (Mao et al. 2015; Cui et al. 2016). SNPs with *P value* ≤ 0.001 in the GWAS study were selected for haplotype detection and further haplotype trait regression. Expectation maximization (EM) algorithm (Excoffier and Slatkin 1995) with 50 EM iterations, EM convergence tolerance of 0.0001 and a frequency threshold of 0.01 were used to estimate haplotype frequency as applied in SVS Version 8.6.0. Block defining algorithm (Gabriel et al. 2002) was used to identify haplotype blocks to minimize historical recombination. Regression analysis was carried out with the haplotype blocks identified on the BLUP values of FSR disease scores, based on step-wise regression with forward elimination.

### Linkage map construction and quantitative trait loci mapping

Linkage maps were constructed using QTL IciMapping version 3.4 software using Kosambi method for map distance calculations. LOD threshold of 3.0 and maximum distance of 40 cM between the two loci were used to detect the linkage between two markers. 158 SNPs from FSR-1 population with 256 F_3_ families and 114 from FSR-2 with 166 F_3_ families were polymorphic between their respective parental lines and were used for constructing the linkage map. BLUP values of disease severity scores of FSR on 1-9 scale were used in QTL analysis. The walking steps in QTL scanning was 1cM and LOD threshold of 3.383 and 3.951 were estimated, respectively, in FSR-1and FSR-2 populations based on 1,000 times permutations analysis (Churchill and Doerge 1994). QTL statistics were also reported for QTLs identified at a lower LOD threshold of 2.5. The sign of additive effect of each QTL was used to identify the origin of favourable allele in accordance with Lubberstedt et al. 1997. In this study, the negative sign of the additive effect indicates that the favourable allele is contributed by the stalk rot resistant line and vice versa.

## Results

### Fusarium stalk rot evaluation of CAAM panel

**Fig. 1:**
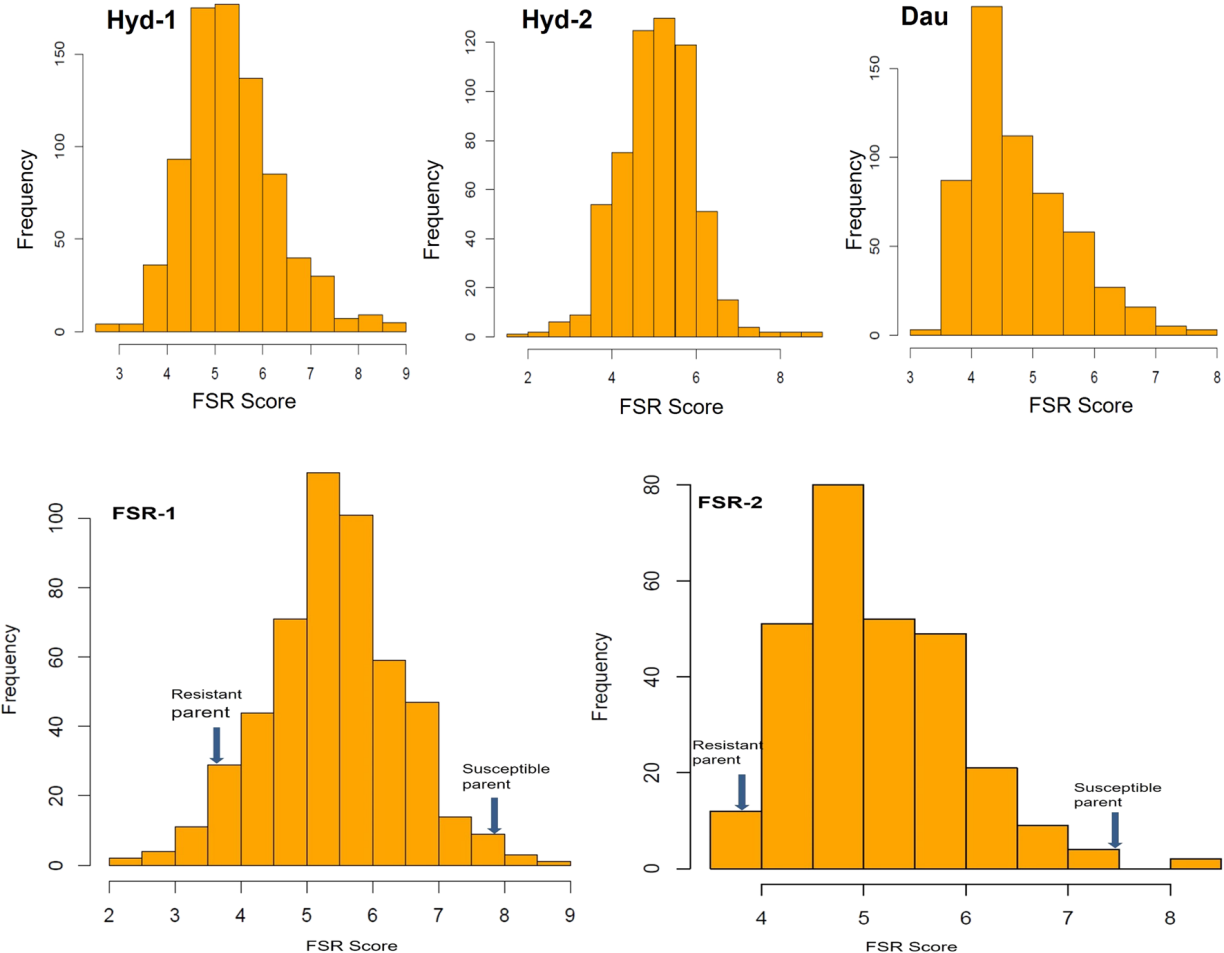
Phenotypic distribution of FSR disease score in CAAM panel evaluated in three environments and two F_2:3_ mapping populations, FSR-1 and FSR-2

A subset of the CAAM panel comprising 342 inbred lines was phenotyped for FSR in three environments under artificial inoculation conditions. Optimal disease expression and high variability was observed during all three years, with maximum disease scores of 8.74, 8.48 and 7.80, and the minimum scores of 3.30, 2.75 and 3.62, respectively, on the disease scale of 1.00 to 9.00 in the trials. Trial means of the individual environments ranged from 4.85 to 5.41. The magnitude of genotypic variance was the lowest (0.18) in Year-3 and the highest in Year-1 (0.41) with an across genotypic variance of 0.14, that was found to be highly significant (*P *value <0.0001). Broad-sense heritability (*h*^2^) estimates were moderate to high, ranging from 0.43 to 0.61, with across trial heritability of 0.51 (Table 1). For QTL mapping, F_2:3_ mapping population FSR-1 showed mean disease score of 5.46 with minimum and maximum score of 3.38 and 7.84. The magnitude of genotypic variance was 0.23 with high significance (*P *value <0.001) and the heritability estimate was 0.40. The second F_2:3_ mapping population FSR-2 showed a trial mean of 5.20 with disease score ranging from 3.89 to 7.57. Highly significant (*P value* <0.0001) genotypic variance was observed, and the heritability estimate was 0.48 (Table 1). The response of the CAAM panel and two F_2:3_ mapping populations showed near-normal distribution for FSR disease severity ranging from resistance to susceptibility (Fig. 1).

**Table 1:** Summary statistics of the CAAM panel and two F_2:3_ mapping populations, FSR-1 and FSR-2 evaluated for FSR under artificial inoculation.

Years	Mean	Min	Max	Phenotypic variance	Error variance	Genotypic variance	G x E variance	Heritability
Hyderabad-1	5.41	3.30	8.74	0.67	0.52	0.41**	–	0.61
Hyderabad-2	5.15	2.75	8.48	0.54	0.52	0.26**	–	0.49
Daulatabad	4.85	3.62	7.81	0.41	0.48	0.18**	–	0.43
Across environments	5.09	3.74	7.82	0.28	0.51	0.14**	0.16**	0.51
FSR-1	5.46	3.38	7.84	0.56	0.67	0.23**	–	0.40
FSR-2	5.20	3.89	7.57	0.37	0.39	0.18*	–	0.48

**P value ≤ 0.0001, G x E, Genotype x environment

### GWAS for Fusarium stalk rot resistance and LD analysis

**Fig. 2:**
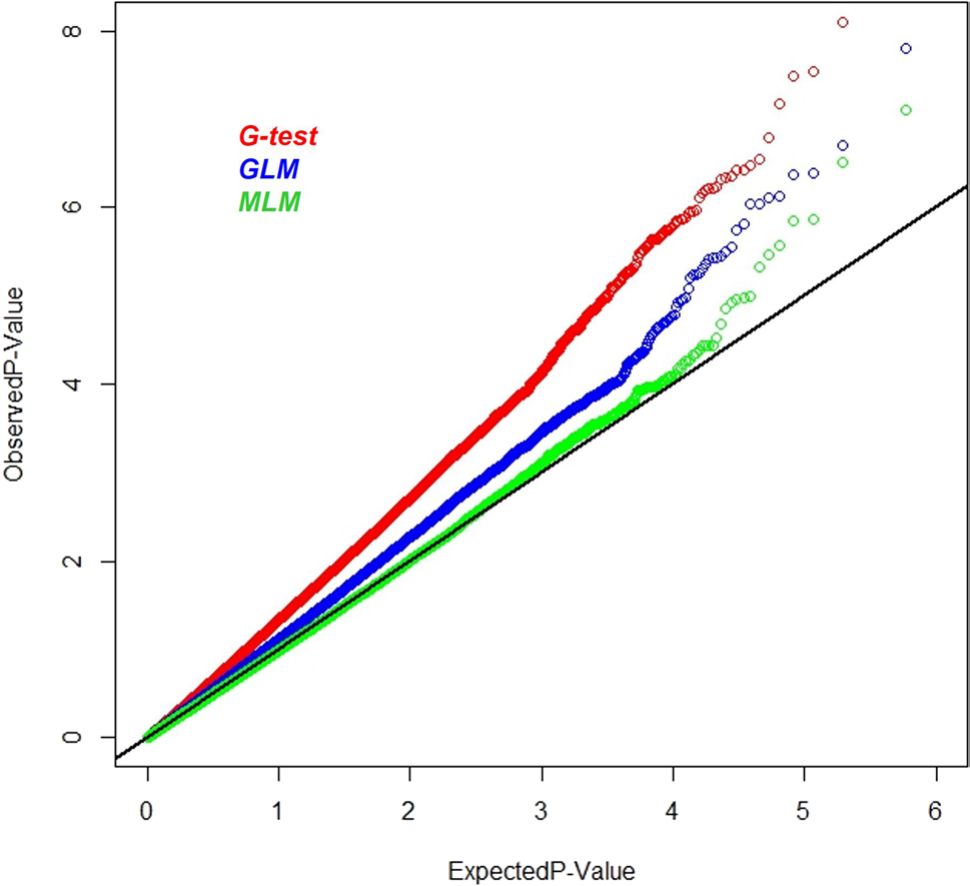
Inflation represented by Q–Q plots of observed versus expected –log_10_ (*P- *values) plots for Fusarium stalk rot using the naïve association model (G-test; red), GLM (G+Q; blue) and MLM (G+Q+K; green); G = genotype (fixed), Q = ten principal components (fixed), K = kinship matrix (random) for CAAM panel

**Fig. 3:**
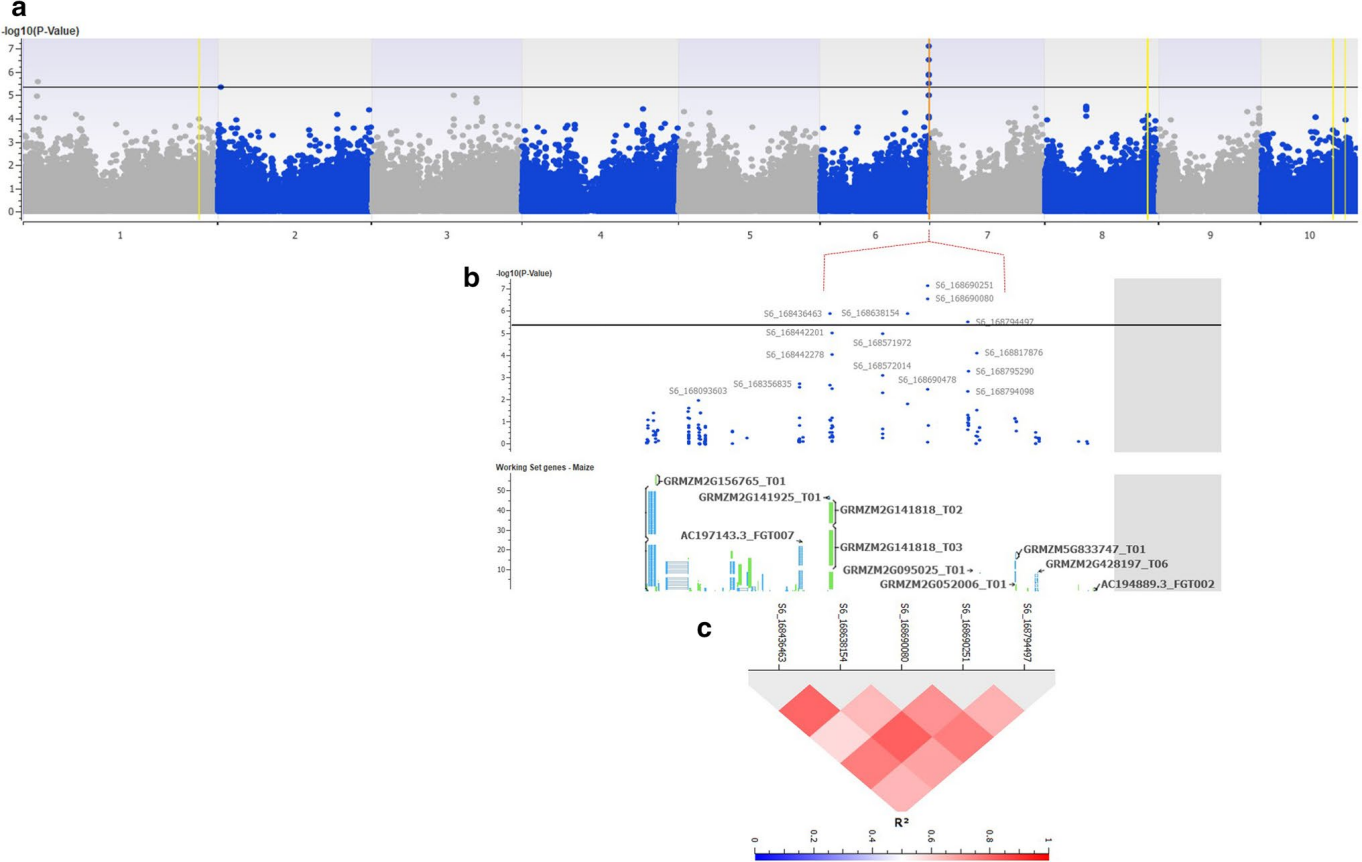
**a** Manhattan plots from GWAS analysis using MLM model, plotted with the individual SNPs on 10 chromosomes on the X-axis and −log_10_ *P*- value of each SNP on the Y-axis. The horizontal line showed the cut-off p value and the vertical lines represent the identified QTLs (orange) and haplotype blocks (yellow) in these regions for Fusarium stalk rot resistance. **b**: Local Manhattan plot at the peak observed on Chromosome 6 between 168.43 and 168.81 Mb. **c**: LD Heat map representing pairwise *r*^2^ values between the SNPs above *P value* threshold at the observed peak on chromosome 6

A subset of 295,794 SNPs, meeting the criteria of call rate ≥0.7 and MAF ≤0.05 from high-density imputed 955K GBS genotyping data, were used for conducting GWAS. Highest genomic inflation was observed in G-test (Naïve model), followed by GLM (G+Q model), which was corrected using 10 principal components. The least genomic inflation was observed in Single locus MLM (G+Q+K) model where the genomic data was corrected for both population structure (Q) and Kinship (K) as detected in QQ plot (Fig. 2). As a result, MLM model was used to discover the significant associations for FSR resistance in the CAAM panel. Seven SNPs were found to be significantly associated with FSR resistance with *P *value ranging from 7.83×10^−8^ to 4.71×10^−6^ on chromosome 1, 2 and 6. Among the seven highly significant SNPs, five SNPs were closely located on Chromosome 6 (S6_168690251, S6_168690080, S6_168638154, S6_168436463 and S6_168794497) at 168Mb (Table 2, Fig. 3a and b). SNP S6_168690251 on chromosome 6 showed the lowest *P value* (7.83×10^-8^) followed by the SNP S6_168690080 (*P *value 3.07×10^−7^). The phenotypic variance explained by the identified significant SNPs in GWAS ranged from 8.38 to 6.16%. In all seven associations identified, the major allele was found accountable for conferring resistance to FSR and the narrow sense heritability with the associated SNPs was found to be 0.61. Significant SNPs detected in GWAS were located within six gene models based on the physical positions of the SNPs with respect to B73 version 2 of reference genome (http://ensembl.gramene.org/Zea_mays). Most of the gene models identified were found to have functional domains involved in tolerance to biotic and abiotic stresses, plant growth and development, resistance to viral and fungal pathogens. LD (r^2^) was estimated in the interval of ~360 Kb that covered the peak on chromosome 6 between 168.43 and 168.79 Mb. Average pairwise *r*^2^ observed for 52 SNPs in this interval was low at *r*^2^ =0.23. The five most significant SNPs on chromosome 6 were in strong LD with an average pairwise *r*^2^ of 0.77 (Fig. 3c)

**Table 2:** List of the highly significant SNPs associated with Fusarium stalk rot resistance in GWAS analysis with the predicted gene models and their functional annotation

Marker	Ch	*P* value	PVE%	Favourable allele	Predicted gene model	Gene name/ best matching ortholog	Functional annotation	Reported function	Plant	References
S6_168690251	6	7.83×10^−8^	8.383	G	GRMZM2G095025	Putative RING zinc finger domain superfamily protein	Ubiquitin protein ligase activity	Plant growth, development and resistance mechanism for Biotic and abiotic stresses	Plants	Gupta et al. (2012), Pi et al. (2018)
S6_168690080	6	3.07×10^−7^	7.645	A	GRMZM2G095025	Putative RING zinc finger domain superfamily protein	Ubiquitin protein ligase activity	Plant growth, development and resistance mechanism for Biotic and abiotic stresses		Gupta et al. (2012), Pi et al. (2018)
S6_168638154	6	1.35×10^−6^	6.840	G	GRMZM2G452452	–	–	–	–	–
S6_168436463	6	1.42×10^−6^	6.815	A	GRMZM2G141818	Argonaute104	Nucleic acid binding	Stress response, anti-viral defence mechanism	Maize, rice Arabidopsis	Zhai et al. (2019), Zhang et al (2015), Qian et al. (2011)
S1_23248968	1	2.65×10^−6^	6.474	G	GRMZM2G005435	–	–	–	–	–
S6_168794497	6	3.37×10^−6^	6.342	G	GRMZM2G052006	Eukaryotic aspartyl protease family protein	Aspartic-type endopeptidase activity	Fungal resistance in plants, biotic and abiotic stress response	Plants, Maize	Li et al. (2016)
S2_4370291	2	4.71×10^−6^	6.161	T	GRMZM2G363066	OSJNBb0022F16.11-like protein	protein serine/threonine kinase activity	Signalling and plant defence, abiotic stresses	Maize, plants	Afzal et al. (2008), Kulik et al. (2011)

### Haplotype regression analysis

Fifty-one haplotype blocks were formed across 10 chromosomes, using the 360 SNPs (*P value* <10^−3^) identified in GWAS, and were used in haplotype regression analysis on the estimated BLUP values of disease scores. HTR analysis detected 32 haplotypes across 9 chromosomes (1, 2, 3, 4, 5, 6, 7, 8 and 10) with Bonferroni *P *value ≤ 0.05, that explained phenotypic variance ranging from 3.75 to 10.91% for FSR resistance. Haplotype Hap_6.3 on chromosome 6 formed by four SNPs (S6_168638154, S6_168690080, S6_168690251, S6_168794497) at 168 Mb had the least Bonferroni *P–*value (3.63×10^−8^) followed by Hap_8.3 on chromosome 8 (Table 3).

**Table 3:** Significant haplotypes detected using haplotype regression analysis for resistance to Fusarium stalk rot in the CAAM panel

Haplotype block name	Ch	SNPs in haplotype block	# Haplotypes	*P* value	PVE%	Bonferroni P	Favourable allele
Hap_6.3	6	S6_168638154, S6_168690080, S6_168690251, S6_168794497	3	3.63×10^−8^	10.913	1.85×10^−6^	AGCC
Hap_6.2	6	S6_168436463, S6_168442201, S6_168442278, S6_168571972, S6_168572014	5	1.10×10^−5^	9.701	0.0005588	TATAC
Hap_1.5	1	S1_285146764, S1_285146770	2	8.20×10^−7^	9.652	4.18×10^−5^	GT
Hap_10.2	10	S10_111787476, S10_111927170, S10_111927184, S10_111927191	3	5.44×10^−6^	9.028	0.0002775	AAAT
Hap_2.1	2	S2_3389145, S2_3389182	3	3.52×10^−6^	8.488	0.0001794	TT
Hap_3.2	3	S3_127191639, S3_127216897	3	9.91×10^−6^	8.439	0.0005053	CT
Hap_8.3	8	S8_159196949, S8_159197240	4	2.51×10^−7^	8.26	1.28×10^−5^	CG
Hap_8.2	8	S8_159196922, S8_159196942	2	3.72×10^−7^	8.008	1.90×10^−5^	GC
Hap_3.1	3	S3_9042320, S3_9042335	2	8.38×10^−6^	7.89	0.0004275	GA
Hap_4.0	4	S4_190681491, S4_190681527	2	7.43×10^−6^	7.418	0.000379	CC
Hap_10.1	10	S10_85192659, S10_85192678	2	7.68×10^−7^	7.148	3.92×10^−5^	GG
Hap_7.1	7	S7_150907723, S7_150959597, S7_150972439, S7_150972510, S7_150972874	3	0.00013	6.912	0.0066075	TCTAC
Hap_6.1	6	S6_130840777, S6_130840880	4	4.89×10^−6^	6.545	0.0002492	CT
Hap_1.6	1	S1_287803582, S1_287803603	3	2.35×10^−6^	6.54	0.0001197	AC
Hap_8.1	8	S8_64086184, S8_64086204, S8_64086226, S8_64086232, S8_64086240	3	1.94×10^−5^	6.522	0.0009913	GCGTC
Hap_8.4	8	S8_171515686, S8_171515699	3	1.25×10^−5^	6.277	0.0006353	TA
Hap_10.3	10	S10_130654033, S10_130654052	2	1.13×10^−5^	6.098	0.0005747	GT
Hap_1.3	1	S1_236292255, S1_236292265	2	1.51×10^−5^	5.642	0.0007679	CG
Hap_7.4	7	S7_163047381, S7_163047383, S7_163047432	2	1.99×10^−5^	5.589	0.0010136	TTA
Hap_6.4	6	S6_168795290, S6_168817876	3	0.000252	5.188	0.0128337	GT
Hap_5.2	5	S5_50451633, S5_50451634	2	7.05×10^−5^	5.138	0.0035931	AG
Hap_5.6	5	S5_178243454, S5_178243456	2	0.000158	4.67	0.008052	TC
Hap_5.1	5	S5_46949044, S5_46949188, S5_46949190, S5_46949191, S5_46949193	3	9.55×10^−5^	4.625	0.0048701	ACTTC
Hap_2.2	2	S2_4465311, S2_4465314	2	8.72×10^−5^	4.538	0.004445	CG
Hap_1.2	1	S1_72704976, S1_72704977	2	0.000618	4.501	0.0315006	AC
Hap_5.3	5	S5_74666936, S5_74666941	2	0.000127	4.387	0.0064527	CC
Hap_1.1	1	S1_25101711, S1_25101740	2	0.000827	4.382	0.0421799	AC
Hap_5.4	5	S5_160457896, S5_160457899, S5_160457902	2	0.000315	4.068	0.0160688	ATT
Hap_5.5	5	S5_178243430, S5_178243432	2	0.000501	3.95	0.0255671	AT
Hap_7.2	7	S7_150973346, S7_150973347, S7_150973348, S7_150973349	2	0.000619	3.8	0.0315614	CCGA
Hap_7.3	7	S7_153838232, S7_153838245	2	0.000701	3.8	0.0357271	TT
Hap_1.4	1	S1_272840349, S1_272840360	3	0.000591	3.754	0.0301598	AT

### Linkage mapping studies for FSR resistance

QTL mapping was carried out in two F_2:3_ bi-parental mapping populations for validation of the genomic regions identified in GWAS and HTR analysis. Linkage maps were constructed by genotyping of two mapping populations, FSR-1 with 158 and FSR-2 with 114 SNP markers that were polymorphic between the parental lines. The average marker density across 10 chromosomes was 6.44 cM and the total map length was 966.20 cM for FSR-1 and for FSR-2 population, the marker density and total map length was 6.47 and 686.12 cM, respectively.

One QTL was identified in FSR-1 on chromosome 7 between PZA00616_13 and PZA02643_1 at 122 Mb and 128 Mb, respectively, based on the physical coordinates of the flanking markers. At a lower LOD threshold of 2.5, two more QTLs were identified in FSR-1 on chromosome 5 and 6. QTL q*FSR*5 detected between the markers PHM13942_9 and PZA00865_on chromosome 5 explained the largest proportion of phenotypic variance (6.531%) in this population. In the FSR-2 population, two QTLs were detected on chromosome 6 and on chromosome 1 at the threshold of LOD 2.5, but no QTL could be identified based on the LOD threshold obtained with 1000 permutations (Table 4). QTL q*FSR*6-1 detected in the confidence interval between the markers PZA02688_2 and PHM3466_69 explained the highest proportion of variance (7.49%) in this population. QTLs q*FSR6* and q*FSR*6*-1* identified on chromosome 6 in FSR-1 and FSR-2, respectively, were found to have one common marker flanking the identified QTLs.

**Table 4:** Quantitative trait loci (QTL) by inclusive composite interval mapping analysis for resistance to Fusarium stalk rot in two F_2:3_ bi-parental mapping populations

Population	QTL	Ch	Ch Bin	Position cM	Marker intervals	Physical position LM	Physical position RM	LOD	PVE (%)	Additive effect	Dominant effect	D/A	Gene action
FSR-1	*qFSR5*	5	5.02–03	52	PHM13942_9-PZA00865_1	21461663	9279777	3.057	6.531	− 0.076	− 0.124	1.625	OD
	*qFSR*6	6	6.07–.08	1	PHM3466_69-PZA00910_1	167148384	166688213	2.522	3.618	0.051	− 0.094	− 1.819	OD
	*qFSR*7	7	7.02	35	PZA00616_13-PZA02643_1	122626201	128365318	4.167	6.391	− 0.11	− 0.01	0.095	A
FSR-2	*qFSR1*	1	1.03	37	PHM3951_25-PZA00240_6	31928634	41231748	2.841	3.970	0.101	− 0.021	− 0.206	A
	*qFSR*6-1	6	6.07–.08	112	PZA02688_2-PHM3466_69	164081101	167148384	3.133	7.49	− 0.154	− 0.048	0.313	PD

## Discussion

*Fusarium verticillioides* is one of the important fungal pathogens causing PFSR of maize. It is a hemibiotrophic pathogen which initiates the disease through roots from soil-borne inoculum and spreads through the nodes and other aerial parts of the plants. FSR is a part of the PFSR complex, where stalks are infected with one or many stalk rot pathogens, which gets exacerbated under abiotic stress conditions like less soil moisture and high usage of fertilizers. PFSR diseases are predicted to either increase or change in pathogen spectrum under the climate change scenario (Prasanna et al. 2021). As a soil-borne disease, use of chemical fungicides is not an efficient option to control the disease. Apart from this, PFSR being a post-flowering disease, it affects the terminal stages of the crop, and hence growing resistant varieties, combining other climate resilience contributing traits is the most sustainable technology to mitigate yield losses from stalk rots in the target geographies in Asia which are climate vulnerable. Breeding efficiency for trait improvement, especially for resistance to biotic stresses, is increased significantly by the inclusion of modern selection tools like molecular markers. CIMMYT and National Agricultural Research System (NARS) partners in India have developed appropriate sick plot-based phenotyping sites for evaluation and identification of stably resistant FSR resistant germplasm for use in breeding pipelines and for developing synthetic populations to increase the frequency of resistant alleles in the breeding pool.

There are very limited studies on trait mapping of resistance to FSR, and considering the importance of this disease in terms of its observed and predicted spread and economic importance, we initiated this study to identify and validate genomic regions associated with FSR resistance using GWAS and linkage mapping. The CAAM panel and two linkage mapping populations were phenotyped for FSR in three and one environments, respectively, under artificial inoculation. The heritability estimates of the trials were moderate (0.40-0.61), suggesting a polygenic nature of the trait, and the results of this study could be used to optimize the most efficient breeding strategy for trait improvement. Genotypic variation was significant in the CAAM panel and the mapping populations, suggesting the superior quality of the phenotypic data that can be used for identification of genomic regions.

Linkage mapping investigates recombination events and marker-trait association in bi-parental segregating populations *viz*, F_2_, double haploid (DH), recombinant inbred lines (RILs), etc. and has an enormous advantage for QTL detection power. Nevertheless, it has limitations like low mapping resolution, limited allele sampling and longer research time. Contrary to linkage mapping, GWAS uses historical recombination from an assembly of lines to analyse marker-trait relationship (Rafalski 2002). It has several advantages over linkage mapping like possibility to use pre-existing populations rather than newly created populations, possibility to survey larger number of alleles, higher mapping resolution and lesser research time (Yu, J. & Buckler 2006; Flint-Garcia, 2005). But considering the advantages of both these mapping methodologies and their complementarity for the purpose of identification and subsequent validation of trait associated/linked genomic regions, both these approaches were used in the present study. The linkage mapping populations were primarily used in this study to validate the identified variants in GWAS for the trait.

The CAAM panel used in this study for GWAS is adapted to tropical and sub-tropical environments and derived from pools and populations developed for tolerance to abiotic stresses like drought, high moisture, heat and nitrogen use efficiency, and resistance to diseases *viz*, downy mildews & blights and insect-pests in Latin America, Sub-Saharan Africa and Asia. A few CIMMYT maize lines (CMLs) released from different CIMMYT regional centres across geographies were also the part of panel. In Asian environments, CAAM panel was earlier used in mapping for resistance to diseases like Sorghum downy mildew, Northern corn leaf blight & Charcoal rot (Rashid et al. 2018, Rashid et al. 2020 and Rashid et al. 2021) and root traits under drought conditions (Zaidi et al. 2016). Two mapping populations were developed by crossing the resistant parents CML578 and CML329/MBRc2amF14-2-B*7 with a common elite, but susceptible parent CML474. These parental lines were part of the CAAM panel and were selected based on their FSR disease responses. These two bi-parental populations were used to validate the genomic regions identified through GWAS for FSR resistance by segregation of relevant alleles at the associated loci (Rafalski 2010) in these populations, apart from identification of novel QTLs that were not detected in GWAS.

GWAS in the CAAM panel identified five highly significant SNPs (S6_168690251, S6_168690080, S6_168638154, S6_168436463 and S6_168794497) associated with FSR resistance on chromosome 6 at 168Mb. Further, haplotype regression test also detected three haplotypes (Hap_6.2 Hap_6.3 and Hap_6.4) having a significant effect on the trait at this genomic region. A clear peak was observed in the Manhattan plot covering the region that spanned 168.43 Mb to 168.79 Mb in the GWAS analysis of which five SNPs which showed the strongest association with the trait were with moderately strong LD with an average pairwise r^2^ value of 0.77. Interestingly, inclusive composite interval mapping also identified QTLs in the two mapping populations on chromosome 6 (bin 6.07-6.08), q*FSR6* in FSR-1 and q*FSR6-1* in FSR-2, albeit with minor effects in these populations. One of the flanking markers, PHM3466_69 at 167 Mb on chromosome 6 was found to be common between these QTLs identified in the two different mapping populations. This region could be of major significance for this trait as it is infrequent to detect stable QTL for complex traits in genetically unrelated populations (Rashid et al. 2021). Further studies are required to dissect this important region to identify the causal variation responsible for this trait. The most significantly associated SNPs S6_168690251, and S6_168690080 were found to be located within the gene model GRMZM2G095025, annotated as a protein with ubiquitin protein ligase activity, which is known for its role in resistance to abiotic & biotic stresses and plant development. E3 Ub-ligases of different families have been shown to be involved in multiple steps of plant immune responses. They are involved in the first step of pathogen response to modulate the perception of pathogen-associated molecular patterns by pattern-recognition receptors and to regulate the accumulation of nucleotide-binding leucine-rich repeat-type intracellular immune receptors (Duplan and Rivas 2014). E3 ligases have also been reported to play important roles in immunity development in rice (You et al. 2016). The rice RING-type E3 OsBBI1 positively regulates the hypersensitive response (HR) and broad-spectrum resistance *against* *Magnaporthe oryzae* (*M.* *oryzae*) (Li et al. 2011). Ring-type E3 is known to be involved in spontaneous programmed cell death (PCD) and non-race-specific resistance to bacterial blight caused by *Xanthomonas oryzae* pv. *oryzae* (*Xoo*) and fungal blast caused by *M.* *oryzae* (You et al. 2016). They are also known to regulate abiotic stress (drought, salinity, heat and cold) responses, through their possible involvement in modifying stress signalling pathways during favourable and unfavourable growth conditions (Lyzenga and Stone, 2021, Melo et al. 2021). SNP S6_168436463, which was also identified as one of the significant SNPs along the same peak observed at 168 Mb on chromosome 6, was located within the predicted gene GRMZM2G141818, which is annotated as a nucleic acid binding protein, Argonaute104. Argonaute (AGO) proteins are RNA binding proteins which play an integral part in gene silencing pathways and many studies reveal the AGO genes respond to diverse abiotic stress conditions in maize (Zhai et al. 2019, Qian et al. 2011). This is especially notable, as FSR disease is known to be aggravated by drought stress. AGO18 family, which is grass specific, has an important role in viral defence and in plant reproductive pathways (Zhang et al. 2015). Another significant SNP in the same genomic region representing the peak at 168 Mb, S6_168794497, is located within the gene GRMZM2G052006 that encodes Eukaryotic aspartyl protease family protein. In *Arabidopsis*, Li et al. 2016 identified a mechanism that couples aspartyl protease with molecular co-chaperone to trigger autophagy and plant defence, providing a key link between fungal recognition and the induction of cell death and resistance. Apart from the significant SNPs identified on chromosome 6, another significant association identified was with SNP S2_4370291 on chromosome 2. This SNP was found to be located in gene GRMZM2G363066 with a functional domain with serine/threonine kinase activity, which are known for their functions in defence mechanism in plants. The receptor serine–threonine kinases are known to interact with other proteins for the processes like diseases resistance, developmental regulation to self- versus non-self-recognition (Goring and Walker 2004) and are also involved in abiotic stresses, abscisic acid-dependent plant development (Kulik et al. 2011).

In studies where genetic variants associated with phenotypic traits are analysed, phenotypic variance explained for any trait increases by using haplotypes for trait regression than single markers, and therefore haplotypes allow the identification of genomic regions responsible for controlling a larger part of variation for the trait of interest (Maldonado et al. 2019). Out of the 32 haplotypes identified in this study that had a significant effect on FSR resistance, some of the haplotypes were found to be co-located in the same chromosomal bins where QTLs/ SNPs have been reported for resistance to other stalk rot pathogens. Zhang et al. 2012 fine-mapped the QTL *qRfg2* for resistance to Gibberella stalk rot (GSR) caused by *Fusarium graminearum* between 257.3 Mb and 277.9 Mb on chromosomal bin 1.10. In this study, we identified a haplotype, Hap_1.4 at 272 Mb, within the physical interval where *qRfg2* was fine-mapped. Similarly, Hap_8.2 and Hap_8.3 identified at 159 Mb in this study were detected within the physical coordinates of the confidence interval of a QTL (146.4-158.9 Mb) for GSR resistance reported by Ma et al. 2017 on chromosomal bin 8.06. Similarly, QTLs/SNPs were identified on chromosomal bin 8.06 for resistance to another PFSR pathogen *Macrophomina phaseolina*, causing Charcoal rot in maize (Rashid et al. 2021). Hence, this region on chromosome 8 could be considered as a region of interest for resistance to multiple stalk rot pathogens as it has been identified for at least three different PFSR diseases. Apart from stalk rots, chromosomal bin 8.06 has been shown to house several QTLs/genes for resistance to other maize diseases like Northern corn leaf blight, Common rust, Common Smut and multiple viral pathogens (Wisser et al. 2005). Another chromosomal region identified for resistance to more than one PFSR pathogens is on chromosomal bin 10.03-04. Two haplotypes identified in our study (Hap_10.1and Hap_10.2) on this chromosomal bin were also found to co-locate with a major QTL *qRfg1* reported for GSR resistance by Yang et al. 2010. These observations from our study point to a possibility that, while there may be genomic regions responsible for imparting resistance to individual stalk rot pathogens, there may be some common regions that could be driving mechanisms for a broad-spectrum resistance towards multiple stalk rot pathogens. Further studies are imperative to throw more light on these observations. With the GWAS and subsequent linkage mapping for FSR resistance in this study, we have identified strong trait marker leads that could further be validated and fine-mapped for deployment in tropical maize breeding programmes to enhance breeding efficiency.

## Conclusion

Genome-wide association studies and linkage mapping were used to investigate the genetic architecture of Fusarium stalk rot resistance in maize. GWAS in a panel of 374 maize lines evaluated for FSR in three environments detected seven highly significant SNPs associated with the trait, of which, five were on chromosome 6 at 168 Mb. Haplotype regression analysis revealed 32 haplotype blocks on chromosome 1, 2, 3, 4, 5, 6, 7, 8 and 10 with significant effect on the trait. Four haplotypes were detected on chromosome 6, of which three haplotypes were located at 168 Mb. For validation of the SNPs identified through GWAS, two F_2:3_ mapping populations FSR-1 and FSR-2 were employed for QTL analysis of FSR resistance. QTLs on chromosome bin 6.07-08 were detected in both the mapping populations with a common flanking marker at 167 Mb. Considering the significance of this region for resistance to FSR, it has to be further studied for fine-mapping and validating in breeding populations for development of deployable trait markers. Interestingly, three genomic regions were identified with haplotypes co-localized with reported QTLs for other stalk rots like GSR and charcoal rot resistance. This raises an interesting proposition of common genomic regions contributing to broad-spectrum stalk rot resistance mechanisms.

## Data Availability

The datasets generated during and/or analysed during the current study are available in the CIMMYT data and software repository network handle https://hdl.handle.net/11529/10548791.
